# Combined repetitive transcranial magnetic stimulation and gut microbiota modulation through the gut–brain axis for prevention and treatment of autism spectrum disorder

**DOI:** 10.3389/fimmu.2024.1341404

**Published:** 2024-02-22

**Authors:** Pengya Feng, Yangyang Zhang, Yonghong Zhao, Pengju Zhao, Enyao Li

**Affiliations:** ^1^Department of Children Rehabilitation, Key Laboratory of Rehabilitation Medicine in Henan, The Fifth Affiliated Hospital of Zhengzhou University, Zhengzhou, Henan, China; ^2^The American Psychiatric Association, Key Laboratory of Helicobacter pylori, Microbiota and Gastrointestinal Cancer of Henan Province, Marshall Medical Research Center, Fifth Affiliated Hospital of Zhengzhou University, Zhengzhou, China

**Keywords:** autism spectrum disorder, rTMS, gut microbiota, gut-brain axis, immunology

## Abstract

Autism spectrum disorder (ASD) encompasses a range of neurodevelopmental conditions characterized by enduring impairments in social communication and interaction together with restricted repetitive behaviors, interests, and activities. No targeted pharmacological or physical interventions are currently available for ASD. However, emerging evidence has indicated a potential association between the development of ASD and dysregulation of the gut-brain axis. Repetitive transcranial magnetic stimulation (rTMS), a noninvasive diagnostic and therapeutic approach, has demonstrated positive outcomes in diverse psychiatric disorders; however, its efficacy in treating ASD and its accompanying gastrointestinal effects, particularly the effects on the gut–brain axis, remain unclear. Hence, this review aimed to thoroughly examine the existing research on the application of rTMS in the treatment of ASD. Additionally, the review explored the interplay between rTMS and the gut microbiota in children with ASD, focusing on the gut-brain axis. Furthermore, the review delved into the integration of rTMS and gut microbiota modulation as a targeted approach for ASD treatment based on recent literature. This review emphasizes the potential synergistic effects of rTMS and gut microbiota interventions, describes the underlying mechanisms, and proposes a potential therapeutic strategy for specific subsets of individuals with ASD.

## 1 Introduction

Autism spectrum disorder (ASD) is a profound neurodevelopmental condition that manifests in infants and young children (1). It is characterized by deficits in social and communication skills and a tendency to engage in repetitive behaviors (2). According to the Global Burden of Disease, Injury and Risk Factor Study 2016, the global prevalence of ASD is estimated at 62.2 million people (3). Moreover, its incidence has evidently increased over time (4). Consequently, it is imperative to prioritize theoretical investigations on and clinical interventions for ASD.

ASD is associated with a wide variety of comorbid conditions such as epilepsy, anxiety, melancholy, Tourette syndrome, and gastrointestinal (GI) disorders (5–7). Among the comorbidities commonly observed in children with ASD, GI issues, including stomach pain, constipation, and diarrhea, have been reported to affect 9%-70% or more individuals (8). The etiology of these GI issues might be associated with alterations in gut microorganisms. In recent years, there has been growing interest in investigating the role of the gut-brain axis in ASD, with studies demonstrating changes in the composition and function of gut microbiota in individuals with ASD. The gut-brain axis refers to the two-way communication between the central nervous system and the individual at large. A recent study indicated that changes in the gut microbiota can affect brain function and development (9). Meanwhile, repetitive transcranial magnetic stimulation (rTMS) has emerged as a noninvasive method of brain stimulation with the potential to ameliorate ASD-related symptoms (10). Research has indicated that rTMS significantly influences the composition and functionality of the gut microbiota (11). Consequently, comprehending the correlation and interplay between rTMS and the gut microbiota is of particular significance for ASD treatment. This article reviews the current literature on the role of rTMS in ASD treatment, underlining its effects on ASD-related gastrointestinal symptoms and exploring potential future implications in ASD treatment.

## 2 Role of rTMS in ASD processing

ASD affects approximately 1 in 59 children, but there are currently no biological treatments that address its underlying symptoms (12). According to preliminary research, rTMS can potentially alleviate the challenges faced by individuals with ASD (13). rTMS is a noninvasive brain stimulation method used to alter brain activity (14). In particular, rTMS under different stimulation modes can lead to long-term changes in activity in target brain regions that possibly outlast the effects of stimulation (15). Substantial research supports the potential efficacy of rTMS in treating the core symptoms of ASD (16).

Several previous studies have identified transcranial direct current stimulation (tDCS) as a promising therapeutic tool for modulating synaptic plasticity abnormalities and minimizing memory and learning deficits in many animal models of neuropsychiatric diseases. The accumulated studies suggest that tDCS modulates brain plasticity via synaptic modifications within the stimulated area. In these studies, changes in plasticity-related mechanisms were achieved through the induction of long-term potentiation (LTP) and upregulation of neuroplasticity-related proteins, such as c-Fos, brain-derived neurotrophic factor (BDNF), or N-methyl-D-aspartate receptors (NMDARs) (17). Moreover, three sessions of tDCS conducted at 1-3-week intervals significantly reduced the levels of several inflammatory cytokines in the brains of healthy rats. The tDCS-mediated reductions in inflammatory cytokine levels highlight its potential use as a countermeasure against inflammation and support the hypothesis that cytokines contribute to the modulation of synaptic plasticity (18). Excitation/inhibition (E/I) imbalance remains a widely discussed hypothesis in ASD. A previous study provided evidence in favor of the excitation/inhibition imbalance hypothesis in ASD and related neurodevelopmental disorders while revealing that its nature is region-specific with distinct pre- and postsynaptic mechanisms. In the hippocampus, disproportionate expression of gamma-aminobutyric acid (GABA)-A receptor dominates, whereas excessive GABA/glutamate ratios are the hallmark of changes in the prefrontal cortex and striatum (19).

### 2.1 rTMS could acted as an investigational tool in ASD

The ideal stimulation settings must be established to maximize the effectiveness and safety of rTMS for ASD treatment. Passing magnetic pulses, stimulating strength, pulse frequency, and interval time are variables in the stimulation process (20). The best stimulus environment that leads to neurological changes in the human body is the basis of the current rTMS program (21). Various TMS paradigms have been developed, including single-pulse TMS, pairing-pulse TMS, and rTMS, to improve lateral network excitation, inhibitory control, and plasticity. These techniques have been used to study neurological ASD, especially in individuals without intellectual barriers (**Table 1**) (15). The influence of rTMS on the brain and behavior is affected by factors such as the position, strength, frequency, quantity, and duration of stimulation and the specific pathophysiology of the disease being treated (28). The dorsolateral prefrontal cortex is a target region for stimulation to improve irritability, repetitive behaviors, and executive functioning (29). The main symptoms and supplementation of the sports cortex are also designed to improve exercise movement (30). The frontal pelt layer of the inner side improves intelligence, and simulation of the frontal cortex improves voice generation and the hand-eye coordination (31).

**Table 1:** The studies of investigational use of TMS in ASD.

Number	Mean age (Maximum to minimum) (years)	Gender (M/F) (M number)	Country	Site	Intensity	Reported Effects	Reference
32	25 (37 to 11)	M/F 18	USA	L M1	120% RMT	No group difference in degree of corticospinal excitability in response to single static hand stimuli or two person interactive hands.	(22)
36	26 (40 to 15)	M/F 15	USA and China	L and R M1	130% RMT; 115% and 130% AMT	No group difference in RMT. Heterogeneous response to paired pulse TMS in the ASD group.	(23)
19	12 (24 to 8)	M 19	USA	L M1	80% AMT	Positive linear relationship between age and duration of modulation of TBS after effects in children and adolescents with ASD. A subgroup of the ASD participants showed paradoxical facilitation	(24)
35	36 (39 to 16)	M/F 12	USA	L M1	80% AMT	Longer lasting inhibition (suppressed MEP) and greater degree of inhibition (area under the curve) following TBS in ASD. Age did not significantly contribute to the model.	(25)
34	26 (30 to 10)	M/F 25	Switzerland	L M1	120% RMT	No group difference in degree of corticospinal excitability in response to observation of single static hand stimuli. Impaired corticospinal facilitaiton in response to single hand transitive hand actions in the ASD group.	(26)
15	18 (22 to 6)	M/F 11	USA	R M1 (PAS)	Lowest intensity producing average 1mV motor-evoked potential	No LTP-like MEP facilitation in ASD (group difference significant at 60 min). No group difference in response to ppTMS.	(27)

M1, Primary Motor Cortex; RMT, Resting Motor Threshold; AMT, Active Motor Threshold; PAS, Paired Associative Stimulation; MEP, Motor Evoked Potential; LTP, long-term potentiation; ppTMS, Paired Pulse TMS; L M1: Left Primary Motor Cortex; R M1: Right Primary Motor Cortex.

The Food and Drug Administration has approved two separate TMS devices, including high- and low-frequency rTMS systems, for the treatment of ASD (32). Both high-frequency (10-20 Hz) and low-frequency (1-5 Hz) rTMS devices have been examined for possible therapeutic effects against ASD. However, the differential frequency of rTMS resulted in different effects on the direction of long-term adaptation in neural excitability. rTMS at 20 Hz bilaterally increased regional cerebral blood flow in the frontal cortex, and surprisingly, 1-Hz rTMS decreased regional cerebral blood flow only in the contralateral prefrontal cortex (33). This raises the possibility of therapeutic applications that selectively activate or inhibit specific areas of the brain in different neuropsychiatric syndromes using this noninvasive procedure. Low- or high-frequency rTMS can be differentially used to reregulate dysfunctional circuits associated with ASD. To reduce ASD-associated excitement, low frequencies are applied to the left prefrontal cortex. This paradigm is used to study irritability and repeated behavior in patients with ASD, which has significantly improved, resulting in the expected defects of cortical suppression (34). Other studies used low-frequency rTMS to modify the operation of various lateral networks in the prefrontal cortex (35). In other studies, different lateral networks in the frontal cortex were differentially affected by low-frequency rTMS (36). Fecteau found that the 1-Hz rTMS can improve naming skills in individuals with ASD (37). When the object is named the left pars opercularis, it is enhanced when the left pars triangularis is applied. Enticott et al. observed improvement of movement-related cortical potentials following rTMS. A single cycle of stimulation of the primary motor cortex and supplementary motor area resulted in increased activity in these areas in subjects with ASD compared to the effects of sham treatment. There was no obvious change in exercise (38). To improve the excitement of the invalid pelt area and the ASD connection, some studies have adopted high-frequency rTMS technology. In double-blind, randomized, placebo-controlled trials, Enticott et al. provided rTMS or sham stimulation to the lateral prefrontal cortex (38) and found no change in the interpersonal response index but found significant improvement in the Ritvo Autism Asperger Diagnostic Scale (RAADS). Panerai et al. utilized high-frequency (8 Hz) stimulation in children with ASD and intellectual difficulties (39) and achieved significant improvement in eye coordination through providing training in behavior hand-fusion.

### 2.2 rTMS could acted as a therapeutic intervention in ASD

In recent years, guidelines for evaluating the therapeutic efficacy of rTMS in a variety of diseases have been published (**Table 2**). Four different types of studies were listed. A prospective, randomized, placebo-controlled clinical trial with concealed outcome assessment and a sample size of at least 25 actively treated patients was considered Class I if the following criteria were met: (a) well-explained dropouts and a sufficiently low number of crossovers to have a minimal likelihood of bias, (b) well-specified exclusion/inclusion criteria, (c) well-defined primary outcome, (d) appropriate consideration of dropouts and crossovers, and (e) the use of correlation analysis. Studies with smaller sample sizes (10-25) or randomized, placebo-controlled studies that did not meet at least one of the aforementioned criteria were considered type II studies. Category III included all other controlled trials. Type IV included uncontrolled studies, case series, and case reports. Although rTMS was the focus of 15 studies and case reports, most of these studies were categorized into Class III or IV. Consequently, the use of rTMS in ASD treatment was classified as Level C (possibly effective) (44).

**Table 2:** The studies of therapeutic use of TMS in ASD.

Number	Mean age (Maximum to minimum) (years)	Gender (M/F) (M number)	Country	Site	Coil	Intensity	Reported Effects	Reference
10	37 (46 to 22)	M/F 6	France, Netherlands, Switzerland, Italy, Serbia, Germany, Finland, Portugal, Monaco	L & R pars opercularis, L & R par triangularis (MRIneuronavigation), sham (central lobe midline)	F08	70% of stimulator output	Increased response latency after L pars opercularis Decreased response latency after L pars triangularis	(37)
45 (25 rTMS, 20 waitlist)	13 (16 to 5)	M/F 38	USA	L & R dlPFC (5 cm anterior to M1)	F08	90% RMT;	Reduced error rate Increased frontal EEG N200 to targets.	(40)
11	18 (21 to 10)	M/F 4	USA	SMA (15% of nasion to inion anterior to Cz), L M1,(Sham M1)	F08	100% RMT	SMA: increased early EEG component PMC: increased EEG negative slope	(38)
1	42	F 0	Italy	M1	–	–	ABC Irritability: Active 40 to 33, Sham 39 to 35, ABC Sterotypy: Active 18 to 12, Sham 16 to 15	(41)
40 (20 rTMS, 20 waitlist)	14 (18 to 7)	M/F 23	China	L & R dlPFC (5 cm anterior to M1)	F08	90% RMT	Reduced error rate Increased frontal EEG P50 amplitude to targets Increased frontal EEG P50 latency to targets	(42)
28 (15 active, 13 sham)	33 (42 to 27)	M/F 18	Italy	dmPFC (7 cm anterior to M1)	H-coil	100%RMT	Reduced social relatedness (RAADS) Reduced personal distress (IRI)	(39)
18	13 (23 to 7)	M/F 10	USA	L & R dlPFC (5 cm anterior to M1)	F08	90%RMT	Increase in R-R interval, SDNN, and HF power. Significant decrease in the LF/HF ratio and SCL. Significant improvements in RBS-R and ABC rating scores	(43)

dlPFC, Dorsolateral Prefrontal Cortex; Fo8, Figure of 8 coil; dmPFC, Dorsomedial Prefrontal Cortex.

Many knowledge gaps remain regarding the use of TMS for ASD treatment, including the stimulus intensity, optimal stimulus settings and application positions, possible clinical goals, and standards for selecting participants. Only a small number of clinical trials (n=4), three of which were randomized, double-blind, placebo-controlled studies (ClinicalTrials.gov IDs NCT02311751, NCT01388179, and NCT00808782), used rTMS as an intervention for different ASD symptom objectives. Before the off-label clinical use of rTMS in the treatment of ASD without an experimental device exemption and outside of an IRB-sanctioned research study can be considered, additional well-controlled trials with adequate power are needed.

## 3 Relationship between the gut microbiota and ASD

**Figure 1:**
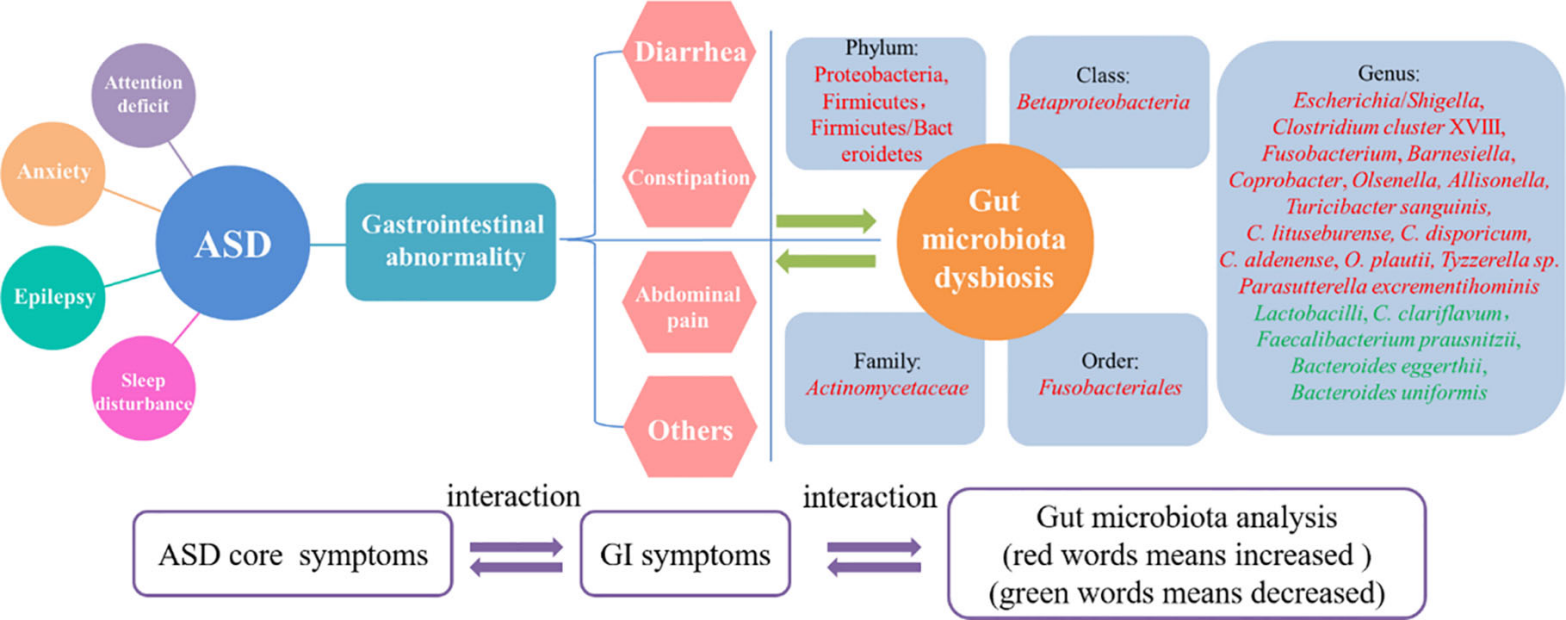
The interrelationship between core symptoms of ASD, gastrointestinal abnormities and gut microbiota dysbiosis. Gastrointestinal abnormities was recognized as one of the core symptoms of ASD as well as anxiety, epilepsy and attention deficit. The mainly features of the gastrointestinal abnormities including diarrhea, constipation and abdominal pain. All these features could resulted in gut microbiota dysbiosis, which showed some pathogenic genus like *Shigella* and *Clostridium cluster* XVIII was increased and some beneficial genus like *Lactobacilli* and *Bacteroides eggerthii* was decreased. The three parts of core symptoms, gastrointestinal symptoms and gut microbiota changes was interacted and mutual influence in ASD.

Numerous studies have revealed that GI issues, such as diarrhea, constipation, and abdominal pain, are common comorbidities in children with ASD and that they are associated with changes in the composition and diversity of the gut microbiota in those with ASD compared to typically developing individuals (**Figure 1**).

### 3.1 Gastrointestinal abnormality correlated with symptom severity of ASD

The occurrence of certain behavior problems is typically used to diagnose GI diseases (45). According to universal reports, there is a great correlation between the severity of ASD symptoms as measured by the Autism Treatment Evaluation Checklist and GI symptoms as measured by the 6-item Gastrointestinal Severity Index (46). The most common GI symptom in children with autism is constipation (47). Abdominal discomfort is significantly related to the severity of ASD core symptoms (48). The aforementioned research assumes that there is an association between GI tract abnormalities and behavior in ASD (49). Additionally, the abnormal GI tract is related to other ASD complications, including sleep problems, strange moods, and social deficiencies. GI comorbidities were found to be associated with sleep problems; mood abnormalities; argumentativeness; oppositional, defiant, or disruptive behavior; anxiety; sensory reactivity; rigidity; obsessive-compulsive behavior; self-mutilation, aggression; lack of expressive language; and social impairment (50).

### 3.2 Gut microbiota dysbiosis is associated with ASD-related gastrointestinal symptoms

Numerous studies revealed that the gut microbiota of children with ASD who experience constipation is significantly different from that of typically developing children. Substantial evidence has showed ASD children with constipation have higher relative abundances of *Escherichia*/*Shigella* and *Clostridium cluster* XVIII (51), the order *Fusobacteriales*, the family *Actinomycetaceae*, and the genera *Fusobacterium*, *Barnesiella*, *Coprobacter*, *Olsenella* and *Allisonella* (52), as well as lower *Faecalibacterium prausnitzii*, *Bacteroides eggerthii*, *Bacteroides uniformis*, *Oscillospiraplautii*, and *Clostridium clariflavum* amount (51). Additionally, although chronic constipation in healthy children is related to fatigue, low lactate levels (53) could explain constipation in patients with ASD (54). In the feces of patients with ASD and allergy, the relative abundance of *Proteobacteria* related to autoimmune diseases is higher (55). Furthermore, an increase in the rate of food allergies has been linked to the ratio of corporate/sterilization. There is an association between ileum and cecum richest company and cecal *Proteobacteria* counts (56). Accumulating evidence has revealed a relationship among feces, immune function, and the corporate/fungus ratio in children with ASD (55). Among children with ASD who report stomach discomfort, *Clostridium aldenense* and *O. plautii* counts are elevated. Some bacteria, such as *C. aldenense* and *O. plautii*, are related to ASD as well as constipation and other GI symptoms (57). Interestingly, among some young individuals with ASD, a Sanguinis related to GI issues was found (58). Parracho et al. proved that children with ASD exhibited higher levels of tissue-soluble toxin producers in their feces, although their levels were not higher than those observed in their healthy siblings (59). High-level scatterro bacteria is closely related to GI problems in individuals with ASD.

### 3.3 Gut microbiota dysbiosis leads to immune system dysregulation in ASD

Gut microbiota dysbiosis in ASD might contribute to immune dysregulation, GI symptoms, and abnormal neurotransmitter metabolism (60). Immune system abnormalities are often caused by gut microbiota dysbiosis in autism (61). A functioning immune system releases chemokines and cytokines including interleukin (IL)-1, IL-6, interferon, and tumor necrosis factor, which can cross the blood-brain barrier. These mediators bind to endothelial cells in the brain and induce immune responses (62). A previous study revealed that the plasma levels of IL-1, IL-6, and IL-8 were considerably higher in patients with ASD than in typically developing controls (63). Additionally, approximately 80% of the immune system is located in and around the gut mucosa (64).

Moreover, the gut microbiota alters brain functioning mostly via its special metabolites. Patients with ASD exhibit increased levels of metabolites including short-chain fatty acids (SCFAs), p-cresol, and ammonia in serum, urine, and feces. These compounds can cause behavioral symptoms in autism and symptoms resembling autism through the vagal pathway (65). Among these metabolites, SCFAs, including acetic acid, propionic acid, butyric acid, valeric acid, and caproic acid, have been identified as major signaling metabolites, playing critical roles in regulating catecholamine production throughout life and preserving the neurotransmitter phenotype after birth (66). These SCFAs were demonstrated by several studies to have importance in ASD (67).

## 4 Potential synergistic effects and mechanism of combined rTMS and gut microbiota modulation in ASD prevent and therapy

**Figure 2:**
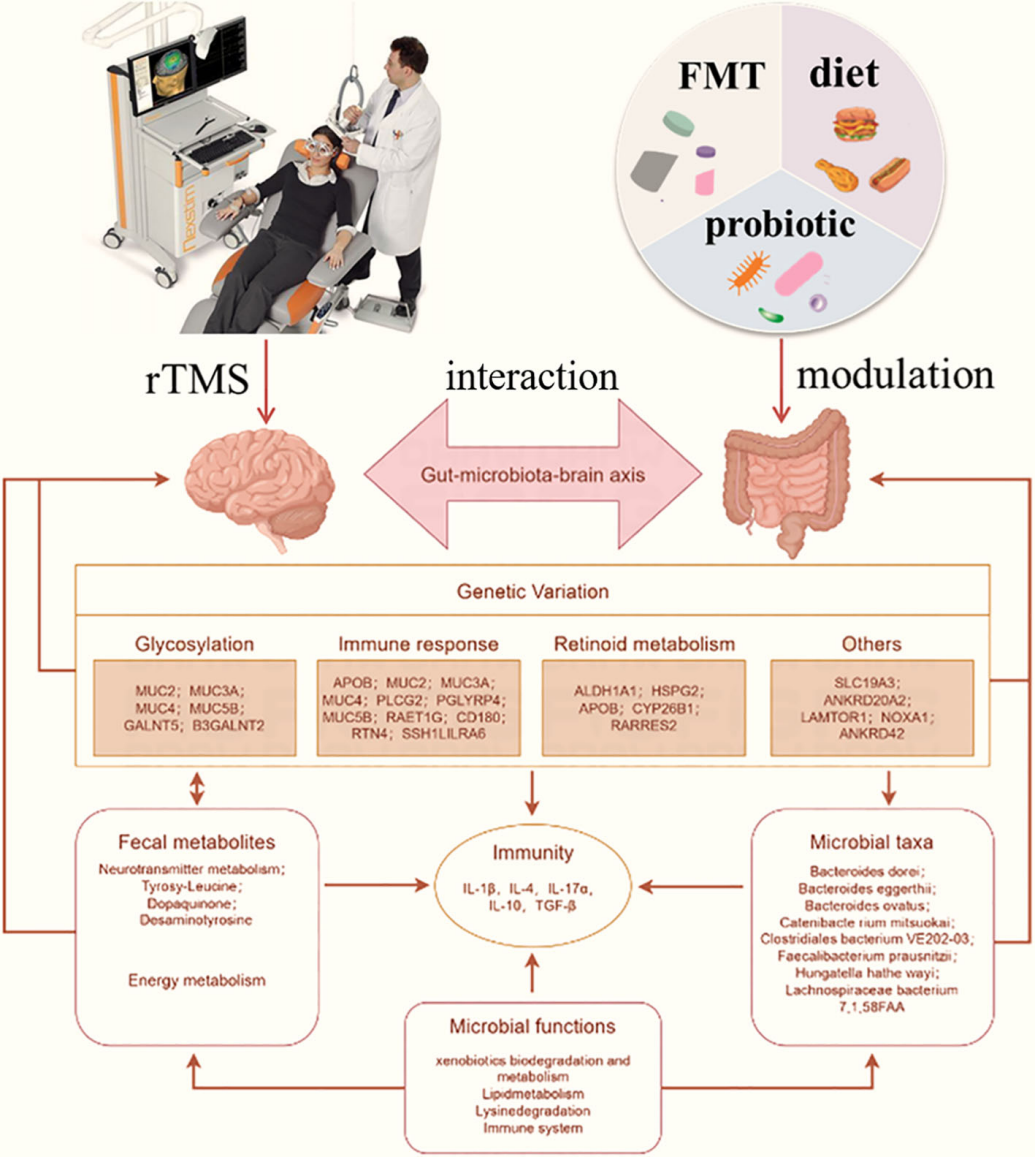
Potential treatment responses triggered by combined rTMS and gut microbiota modulation against ASD through interaction by gut-brain axis. rTMS can directly affect nervous function like glycosylation, immune response and retinoid metabolism to further affect brain performance. The fecal metabolites and microbial taxa was affected by gut microbiota modulation through diet, probiotics or FMT, which was also affect the immunity like IL-4, IL-10 and TGF-β. The interaction between microbial and nervous was achieved by gut microbiota-brain axis, which the intestinal function and brain function was mutual influence. Combination rTMS and gut microbiota modulation can play a double regulation that is contributed to the treatment of ASD.

Poor social interaction, communication problems, and repetitive behaviors are hallmarks of ASD, which is a complex neurodevelopmental disorder (68). Numerous studies suggest that the development of autism depends on both hereditary and environmental factors (69). rTMS, as a noninvasive brain stimulation technology, has gained popularity and exhibited potential to treat ASD symptoms (70). Interest in the role of the gut-brain axis in ASD has increased recently because of studies demonstrating a relationship between gut microbiota composition and function in children with ASD. Thus, rTMS and gut microbiota modulation could be used as a combined treatment for ASD (**Figure 2**).

A previous study demonstrated the existence of a sophisticated network of nervous, metabolic, immune, and other associations between the brain and the GI tract, known as the gut-brain-microbiome axis (71). rTMS influenced selected phyla of the gut microbiota, and the changes were associated with alterations in behavioral and metabolic parameters. Recent investigations suggest that alterations of the composition and diversity of gut microbiota caused by impaired energy homeostasis could play an important role in the development of neuropsychiatric and metabolic disorders (72). Furthermore, rTMS can reverse adverse effects on the gut microbiome, supporting the hypothesis of the existence of complex bidirectional communication between the brain and GI tract (gut-brain-microbiome axis). Several pathways, including those involving the vagus nerve, the immune and endocrine system, and the enteric nervous system, mediate the bidirectional communication between the brain and gut microbiota (73).

### 4.1 Combination of rTMS and gut microbiota modulation could modulate the nervous system

rTMS can enhance neuroplasticity by modulating neural activity and promoting synaptic plasticity (74). Some protocols appear to induce suppression or facilitation through Hebbian mechanisms of long-term depression and long-term potentiation across populations of neurons, whereas others induce these changes by modulating activity in GABAergic interneurons. The prominent role of inhibitory interneurons in the rTMS-induced modulation of cortical excitation is of importance in autism, similarly as the GABAergic system. rTMS as an investigational treatment partially supports the theories suggesting excitation/inhibition imbalance and aberrant plasticity mechanisms in ASD. Conversely, gut microbiota modulation can influence neuroplasticity through the production of neuroactive compounds and regulation of neurotransmitter metabolism (75). By combining these interventions, it is possible to enhance neuroplasticity mechanisms in the brain, leading to improved cognitive and behavioral outcomes in individuals with ASD. Additionally, the modulation of neurotransmitters is another potential pathway explaining the effects of the combined treatment. Both rTMS and gut microbiota modulation can influence neurotransmitter levels in the brain (76, 77). Gamma-aminobutyric acid (GABA) and glutamate are two neurotransmitters modulated by rTMS (78). Through the generation of metabolites including SCFAs and neurotransmitter precursors, gut microbiota modification can also affect neurotransmitter metabolism (79). By combining these interventions, it is possible to more comprehensively modulate neurotransmitter levels, which help alleviate ASD symptoms related to neurotransmitter dysregulation.

### 4.2 Combination of rTMS and gut microbiota modulation could regulate the immune response

**Figure 3:**
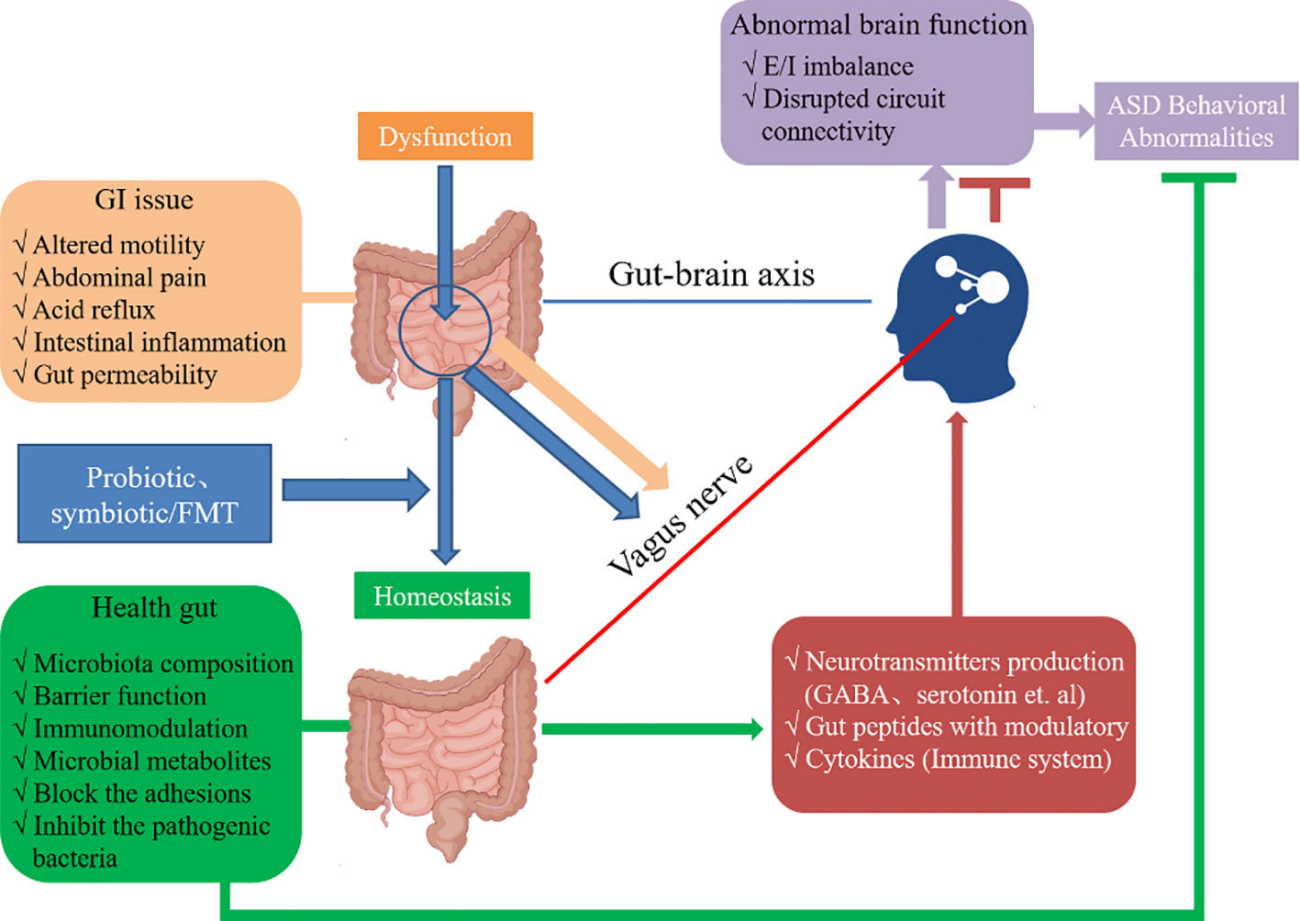
The ASD and dysregulation within the gut-brain axis and the appropriate mechanisms of interplay. The probiotics (e.g., *Lactobacillus*), symbiotics or FMT can enhance the homeostasis to maintain intestinal health. The microbiota composition and production of metabolites such as SCFAs can cross the “leaky gut” to affect brain function. Moreover, some microbiota can produce neuroactive compounds (e.g., GABA) that cross the “leaky gut” and influence brain function and induce abnormal behaviors. These neuroactive compounds can directly influence the HPA axis and increase circulating levels of cortisol. Metabolites, certain microbiota and neuroactive compounds can activate enteric neurons and affect brain function through the vagus nerve. Some microbiota and metabolites can activate gut immune cells, which can release cytokines into circulation.

rTMS and gut microbiota modulation have been implicated in the regulation of inflammation (80, 81). Inflammation is known to participate in the pathophysiology of ASD, and reducing inflammation can potentially alleviate symptoms associated with the disorder (82). rTMS can modulate the immune response and reduce pro-inflammatory cytokine levels (83). Gut microbiota modulation can also regulate inflammation through the production of anti-inflammatory factors and modulation of immune cells (83). By combining these interventions, it is possible to achieve synergistic anti-inflammatory effects, which might help alleviate ASD symptoms. Bidirectional communication between the gut and brain is achieved through the gut-brain axis, which plays a crucial role in the regulation of brain function and behavior. Both rTMS and gut microbiota modulation can influence the gut-brain axis (84, 85). rTMS can modulate gut microbiota composition and function through its effects on neurotransmitters and the immune system (86). Conversely, gut microbiota modulation can affect brain function and behavior through the production of neuroactive compounds, modulation of the immune system, and regulation of the intestinal barrier (87). By combining these interventions, it is possible to more comprehensively regulate the gut-brain axis, thereby improving ASD symptoms (**Figure 3**).

### 4.3 Combination of rTMS and gut microbiota modulation could modulate psychological aspects

The potential synergistic effects of the combination of rTMS and gut microbiota modulation in ASD can be explored in other areas. GI symptoms, including abdominal pain, constipation, and diarrhea, are commonly reported in individuals with ASD (88). Gut microbiota modulation has been demonstrated to improve GI symptoms by restoring the balance of gut bacteria and reducing gut inflammation (89). rTMS, through its effects on the gut-brain axis, might also have a positive impact on GI symptoms. By combining these interventions, it is possible to target both the neurological and gastrointestinal aspects of ASD, leading to a more comprehensive improvement in overall well-being. Anxiety and depression are frequently observed in individuals with ASD (90). Both rTMS and gut microbiota modulation have proven antidepressant and anxiolytic effects. rTMS can modulate neural circuits involved in mood regulation (91), whereas gut microbiota modulation can influence the production of neurotransmitters and neuroactive compounds involved in mood regulation (92). By combining these interventions, it is possible to target both the neurological and psychological aspects of ASD, leading to reductions in anxiety and depression symptoms. Impairments in social communication and interaction are core features of ASD (93). rTMS has been revealed to modulate brain regions involved in social cognition and empathy, which are often impaired in individuals with ASD (94). Gut microbiota modulation can also influence social behavior through its effects on neurotransmitters and the immune system (95). By combining these interventions, it is possible to target the underlying neural mechanisms involved in social communication and interaction, leading to improvements in these domains.

## 5 Conclusion and future directions

In this review, we discussed the interrelationship between rTMS and the gut microbiota via the gut-brain axis in ASD. We also discussed the potential of combining rTMS with gut microbiota modulation as a novel therapeutic strategy for the prevention and treatment of ASD in children. However, further investigation is required to clarify the best treatment approaches, long-term outcomes, and safety profiles of these therapies. Additionally, to develop successful and focused therapies for individuals with ASD, deeper understanding of the underlying processes and the intricate relationships among rTMS, the gut microbiota, and the brain is essential. As we indicated, rTMS is believed to be safe when performed in compliance with current safety standards, particularly in pediatric populations. However, rTMS carries a small risk of unfavorable side effects. Therefore, before patients receive rTMS, several criteria, including their current medicines and medical history, must be evaluated. Additionally, the procedure’s risk-benefit ratio must be carefully considered.

## Author contributions

PF: Conceptualization, Writing – original draft, Writing – review & editing. YYZ: Data curation, Software, Writing – review & editing. YHZ: Investigation, Writing – review & editing. PZ: Writing – review & editing. EL: Writing – review & editing.
